# Supplementary material for: Toward a Common Performance and Effectiveness Terminology for Digital Proximity Tracing Applications
Source: Front Digit Health. 2021 Aug 5;3:677929. doi: 10.3389/fdgth.2021.677929 (PMC8521913; doi:10.3389/fdgth.2021.677929)
Supplement: Supplementary file 1 [file Table_1.docx]

| **Key performance indicator** | **Indicator Type  (Matrix Cells  in Figure 3)** | **Possible Sources** | **Remark** | **Implemented in (examples)** |
| --- | --- | --- | --- | --- |
| Number of app downloads | 3, 4 | Data from App stores (Google, Apple) |  | CH [[1](#_ENREF_1)] DE [[2](#_ENREF_2)], NL [[3](#_ENREF_3)] |
| Number of active apps | 3, 4 | DPT backend | Counted by apps randomly sending “empty requests” to backend system (on average once every 5 days). This measure is a proxy for the number of active app users. | CH [[1](#_ENREF_1)] |
| Number of entered upload authorization codes | 4 | DPT backend |  | CH [[1](#_ENREF_1)], DE [[2](#_ENREF_2)], NL [[3](#_ENREF_3)] |
| Time from symptom onset (as reported by participant) to upload of tracing keys | 4 | DPT backend | Date of positive SARS-CoV-2 test if person is asymptomatic | CH [[1](#_ENREF_1)] |
| Number of hotline calls after exposure notification | 3,4 |  | In Switzerland, hotlines were partially overwhelmed early during the second pandemic wave. Therefore, monitoring calls may also reveal technical and resource bottlenecks. | CH [[1](#_ENREF_1)], DE [[2](#_ENREF_2)] |
| Number of notifications shown to users | 5 (or maybe 3,4) | Statistics provided by apps |  | IT [6] |
| Exposure notification as reason for seeking SARS-CoV-2 testing | 6 | Statistics on tests (e.g. as collected by testing laboratories), population surveys |  | CH [[4](#_ENREF_4)], NL [[3](#_ENREF_3)] |
| Positive SARS-CoV-2 tests after exposure notification | 6 | Statistics on tests (e.g. as collected by testing laboratories), population surveys |  | CH [[4](#_ENREF_4)], NL [[3](#_ENREF_3)] |
| Persons with EN who (self-)quarantine | 6 | Post-EN surveys, calls to hotline, web-based risk-assessment tools |  | CH [[4](#_ENREF_4),[5](#_ENREF_5)] |

**Supplementary Table 1: Examples of Key Performance Indicators**

**Example measures of comparative effectiveness:**

derived from indicators of public health relevance

- Test positivity among persons with an EN, compared to exposed persons without EN [[4](#_ENREF_4),[3](#_ENREF_3)]
- Ratio of number EN-triggered quarantine recommendations over number of MCT-mandated quarantines [[5](#_ENREF_5)]

**Further information on recommended key performance indicator and effectiveness measurements can also be found in a joint World Health Organization and European Center for Disease Control document [7].**

**Abbreviations**: DPT, Digital Proximity Tracing

**References**:

1. Swiss Federal Office of Statistics: SwissCovid App Monitoring. <https://www.experimental.bfs.admin.ch/expstat/de/home/innovative-methoden/swisscovid-app-monitoring.html> (accessed July 19, 2021).

2. Hoerdt, J.: Current facts and figures about the Corona-Warn-App. <https://www.coronawarn.app/en/blog/2021-07-09-facts-and-figures/> (accessed July 19, 2021).

3. Rijksoverheid: Factsheet Coronamelder. <https://coronamelder.nl/media/Factsheet_Corona_latest.pdf> (accessed 21.04.2021)

4. Ballouz, T., Menges, D., Aschmann, H.E., Domenghino, A., Fehr, J.S., Puhan, M.A., von Wyl, V.: Digital proximity tracing app notifications lead to faster quarantine in non-household contacts: results from the Zurich SARS-CoV-2 Cohort Study. International Journal of Public Health (2021). In print.

5. Menges, D., Aschmann, H.E., Moser, A., Althaus, C.L., von Wyl, V.: The role of the SwissCovid digital contact tracing app during the pandemic response: results for the Canton of Zurich. Jama Network Open (2021). 4(4):e218184.

6. Presidenza del Consiglio dei Ministri. <https://www.immuni.italia.it/dashboard.html> (accessed July 19, 2021).

7. World Health Organization & European Centre for Disease Prevention and Control. Indicator framework for the evaluation of the public health effectiveness of digital proximity tracing solutions: <https://apps.who.int/iris/handle/10665/341818> (accessed July 19, 2021).
